# Causal Structural Modeling of Survey Questionnaires via a Bootstrapped Ordinal Bayesian Network Approach

**DOI:** 10.1017/psy.2024.11

**Published:** 2025-01-03

**Authors:** Yang Ni, Su Chen, Zeya Wang

**Affiliations:** 1 Department of Statistics, Texas A&M University, College Station, TX, USA; 2 Center of Transforming Data to Knowledge, Rice University, Houston, TX, USA; 3 Department of Statistics, Rice University, Houston, TX, USA; 4 Dr. Bing Zhang Department of Statistics, University of Kentucky, Lexington, KY, USA

**Keywords:** Causal discovery, directed acyclic graph, survey data, categorical data, identifiability

## Abstract

Survey questionnaires are commonly used by psychologists and social scientists to measure various latent traits of study subjects. Various causal inference methods such as the potential outcome framework and structural equation models have been used to infer causal effects. However, the majority of these methods assume the knowledge of true causal structure, which is unknown for many applications in psychological and social sciences. This calls for alternative causal approaches for analyzing such questionnaire data. Bayesian networks are a promising option as they do not require causal structure to be known *a priori* but learn it objectively from data. Although we have seen some recent successes of using Bayesian networks to discover causality for psychological questionnaire data, their techniques tend to suffer from causal non-identifiability with observational data. In this paper, we propose the use of a state-of-the-art Bayesian network that is proven to be fully identifiable for observational ordinal data. We develop a causal structure learning algorithm based on an asymptotically justified BIC score function, a hill-climbing search strategy, and the bootstrapping technique, which is able to not only identify a unique causal structure but also quantify the associated uncertainty. Using simulation studies, we demonstrate the power of the proposed learning algorithm by comparing it with alternative Bayesian network methods. For illustration, we consider a dataset from a psychological study of the functional relationships among the symptoms of obsessive-compulsive disorder and depression. Without any prior knowledge, the proposed algorithm reveals some plausible causal relationships. This paper is accompanied by a user-friendly open-source R package OrdCD on CRAN.

## 1 Introduction

Survey questionnaires are often used in social, psychological, and behavioral sciences to measure various traits of individuals, which are otherwise hard to assess. For example, Posttraumatic Stress Checklist is often used for measuring post-traumatic stress disorder symptoms, Yale-Brown Obsessive-Compulsive Scale for obsessive compulsive disorder, and Quick Inventory of Depressive Symptomatology for depression, just to name a few.

Various causal inference methods such as the potential outcome framework and structural equation models (SEMs) have been used to infer causal effects.

The potential outcome framework (Neyman, 1923; Rubin, 1974) is a widely used approach to estimate the effects of treatments on outcomes from observational studies. It defines the treatment/causal effect of an experiment unit by contrasting the outcome under the treatment and the outcome under the control. The fundamental challenge of causal inference is that only one of the two potential outcomes can be observed for each experimental unit. Naively using observed outcomes alone to estimate (average) causal effects will be biased due to confounding effects. Properly adjusting for confounders is therefore key to the success of the potential outcome framework.

An SEM refers to a set of stochastic equations describing the statistical causal relationships among observed and latent variables (Jöreskog, 2005; Tarka, 2018). In the psychological field, the latent variables represent latent psychological states or traits, which are believed to exist but difficult to quantify or measure directly, and the observed variables are the “symptoms” or indicators of the latent traits, which, by contrast, can be measured by questionnaires. An SEM is comprised of two components: a measurement/factor model and a structural/path model. The measurement model connects observed variables to latent variables whereas the structural model specifies the relationships among the latent variables, which reflect the causal assumptions made by investigators.

Despite the success of these causal models, alternative causal approaches are called for due to a few prominent limitations. First, both the potential outcome framework and SEMs typically assume the causal relationships among observed/latent variables to be known *a priori*. For example, in the potential outcome framework, one has to know which variables are treatments and which variables are outcomes. However, in many psychological applications, the true causal relationships are unknown, and the inferential results can be quite sensitive with respect to the misspecification of the causal relationships, which could lead to various practical issues such as the Haywood case or the negative variance problem for SEMs (Bentler & Chou, 1987; Kolenikov & Bollen, 2012), and, more seriously, causal effect estimation bias and misinterpretation (Kolenikov, 2011), which are partially responsible for the replicability crisis in psychology and social science and cannot be alleviated by increasing sample size (Vowels, 2021). Second, a latent variable in an SEM often causes multiple symptoms, which implicitly assumes that the symptoms are conditionally independent of each other given the latent variable. However, symptoms can affect each other directly. For example, lack of appetite can cause weight loss and hence they are not independent of each other conditioned on their common cause such as depression. Third, some SEMs can accommodate scenarios where the causal relationships are only partially known. For example, the ordinal SEM (Luo et al., 2021, OSEM) learns the causal structure among latent variables from the data. However, the causal structure is not uniquely identifiable (i.e., multiple causal structures can fit the data equally well), and, therefore, no definitive conclusion can be drawn from such methods.

An alternative class of models for causal analyses is Bayesian networks Pearl (1988), which can overcome the aforementioned limitations of the potential outcome framework and SEMs because BNs typically do not assume the underlying causal structure to be known *a priori*. BNs are a type of probabilistic graphical model that can be used to represent and learn causal relationships of a set of variables in an unbiased, objective, data-driven way. Many fields of science, such as neuroscience (Shen et al., 2020), climate science (Ebert-Uphoff & Deng, 2012), and robotics (Lazkano et al., 2007), have seen rapidly growing enthusiasm for using BNs to discover unknown causal structures. For example, in systems biology, BNs have been shown to successfully recover gene regulatory networks from observational, cross-sectional genomic data without any prior biological knowledge (Chai et al., 2014; Choi et al., 2020; Choi & Ni, 2023; Friedman et al., 2000; Sachs et al., 2005; Zhou et al., 2023).

The potential of BNs to characterize complex causal relationships for survey data in social and behavior sciences has, however, only been demonstrated in a few recent works (Bird et al., 2019; Briganti, 2022; Briganti et al., 2022; Fried et al., 2017; Luo et al., 2021; McNally et al., 2017). Although they already provided compelling evidence that BNs are powerful causal analysis tools, which complement the potential outcome framework and SEMs, their techniques tend to suffer from causal non-identifiability with observational data. For example, Bird et al. (2019) wrote “as distinct causal models can lead to the same patterns, it is not possible to learn all the causal links from observational data.” However, since the seminal paper (Shimizu et al., 2006) published in 2006, numerous BNs (e.g., Hoyer et al., 2009; Zhang & Hyvärinen, 2009) have been proven to be fully identifiable under various assumptions. Most relevant to the survey questionnaire data is the recent development of ordinal BNs (Ni & Mallick, 2022). They theoretically proved that the causal structure is fully identifiable by exploiting the ordinal nature of categorical data, which had not been thought to be important for causal discovery, and empirically validated it with multiple real datasets such as discretized protein expression data.

Furthermore, non-identifiable BNs such as the commonly used categorical BNs can lead to unintended negative consequences if one is not careful in interpreting the inferred causal networks. Because multiple causal networks can fit the data equally well for non-identifiable BNs, it would be generally incorrect to interpret the causal relationships from a single causal network.

In this paper, we advocate the use of ordinal BNs in social and behavior sciences as a lot of questionnaire data collected are naturally ordinal. We develop a causal structure learning algorithm with bootstrapping, which aims to identify optimal causal structures with finite-sample uncertainty quantification and large-sample guarantee. Using simulation studies, we demonstrate the power of the proposed learning algorithm by comparing it with competing BNs. Subsequently, we apply the ordinal BNs to a dataset of obsessive-compulsive disorder and depression, which reveals some plausible causal relationships without resorting to any prior knowledge. For reproducibility and broad applicability, we make a user-friendly R package [name hidden] freely available on CRAN.

Our main contributions are four-fold:

1. We develop a new causal structure learning algorithm with uncertainty quantification.
2. We make a new user-friendly R package available to the scientific community.
3. We introduce a novel application of ordinal BNs to psychological survey data.
4. We provide an asymptotic justification of our method, which guarantees the correctness of the estimated causal graph for a large enough sample size.

## 2 Overview of probabilistic graphical models

Let 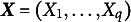 denote a vector of *q* random variables. For questionnaire data, 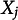 represents the available choices of question *j*, e.g., 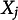 may take value from “Strongly Disagree”, “Disagree”, “Neutral”, “Agree”, and “Strongly Agree” for a 5-point Likert scale question. For convenience, 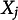 is often coded numerically, e.g., 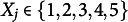 ; however, note that the actual number does not have an absolute interpretation but its relative ordering is informative in the sense that 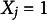 is closer to 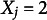 than to 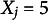 . To represent the (causal or non-causal) dependencies among a set of random variables, probabilistic graphical models are often used. Let 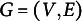 denote a graph with a set of nodes 
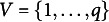
 corresponding to the random variables 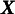 and a set of edges *E* representing the dependencies. The type of edges that the edge set *E* contains dictates the type of dependencies that a graph can represent. We will restrict our discussion to two commonly used types of graphs, undirected graphs and directed acyclic graphs, with a focus on the latter.

### 2.1 Undirected graphs

The edge set *E* of an undirected graph contains only non-directional edges 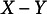 , which are useful for representing symmetric associations. The presence (absence) of an edge between two variables indicates a statistically significant marginal correlation or partial correlation (the lack thereof). For ordinal variables, the polychoric correlation may be used. Partial correlation is often deemed more appropriate than marginal correlation as partial correlation is a measure of conditional dependence accounting for all the other variables of interest and, therefore, can avoid detecting spurious indirect association from marginal correlation. However, by design, undirected graphs based on marginal or partial correlation cannot be used to represent causal relationships, which are asymmetric and directional.

### 2.2 Directed acyclic graphs and Bayesian networks

**Figure 1:**
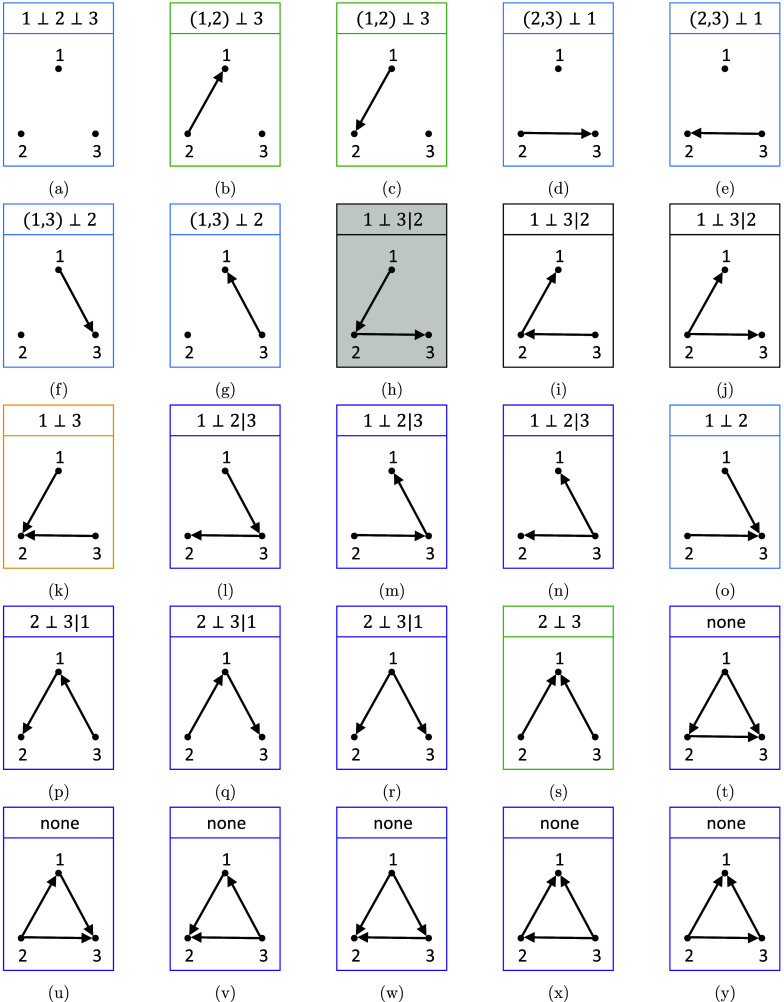
All possible three-node DAGs. The conditional independence assertion encoded by each graph is shown at the top of each DAG.

The edge set *E* of a directed acyclic graph (DAG) contains only directed edges or arrows 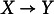 . In addition, we assume that there is no directed cycle, i.e., one cannot return to the same node by following the arrows. A DAG, by itself, is a pure mathematical object, which needs to be connected to data through probability models. The most well-known probability model of such kind is the *Bayesian network* (BN) proposed by (Pearl, 1988). A BN is a pair 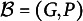 where *G* is a DAG and *P* is a probability distribution that is linked to the DAG *G* through the BN factorization, (1)

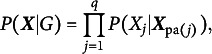

where 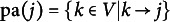 is the *parent* set of node *j* and 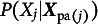 is the conditional probability distribution of node *j* given its parents. For categorical variables, 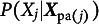 is conditionally multinomial, typically specified by a conditional probability table. For example, if 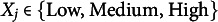 is an employee’s pay grade and 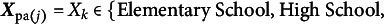 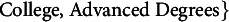 is the employee’s education level, then 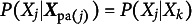 can be specified by a 3 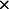 4 conditional probability table with the first row and second column being the probability 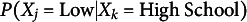 that an employee is at the low pay grade if his/her highest degree is high school. The BN factorization (1) implies a set of conditional independence assertions, also known as the Markov property, which can be directly read off from *G* (Lauritzen, 1996). For instance, the probability distribution *P* must respect the following conditional independence (known as the *local Markov property*): any variable is conditionally independent of its non-descendants given its parents, 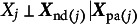 where 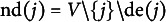 denotes the set of non-descendants of node *j* with 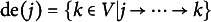 being the set of descendants of node *j*. Importantly, the reverse is also true, i.e., if a distribution *P* satisfies the local Markov property of a DAG *G*, it must factorize with respect to *G* as in (1). For example, for the three-node DAG (h) in Figure 1 where, say, 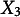 is an employee’s pay grade, 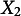 is the employee’s education level, and 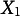 is the education level of the employee’s mother, specifying the joint distribution of 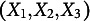 through the conditional distribution 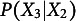 of the employee’s pay grade given his/her education level, the conditional distribution 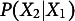 of the employee’s education level given his/her mother’s education level, and the marginal distribution 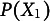 of the education level of the employee’s mother is equivalent to assuming that the employee’s pay grade is independent of the education level of the employee’s mother given the employee’s education level.

### 2.3 Causal DAGs and causal BNs

The arrows of a DAG do not have physical interpretations and, consequently, a BN is merely a probability model that encodes certain conditional independence assertions and factorizes in a certain fashion with respect to its associated DAG. To equip BNs with causal interpretations, we need to first define a causal DAG. A causal DAG is a DAG for which the directed edges are causal. For example, DAG (h) in Figure 1 means that node 1 (e.g., blood pressure) is a direct cause of node 2 (e.g., heart attack), which is in turn a direct cause of node 3 (e.g., death), and node 1 is not a direct cause of node 3. Then we assume a probability distribution *P* is *causal Markov* with respect to *G*, i.e., any variable is conditionally independent of its non-effects given its direct causes, 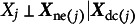 where 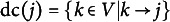 is the set of direct causes of *j* and 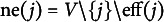 is the set of non-effects of *j* with 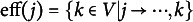 . For instance, in DAG (h), death is conditionally independent of blood pressure given the patient has heart attack (although in real life, abnormal blood pressure can cause death in many ways other than heart attack but the missing arrow between nodes 1 and 3 in DAG (h) excludes alternative causal paths between blood pressure and death in this illustrative example).

Noticing the equivalence in definition between the parents 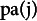 and the direct causes 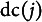 , and between the non-descendants 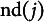 and the non-effects 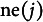 , one can immediately conclude that the probability distribution *P* must factorize with respect to the causal DAG *G* as in (1) given the causal Markov assumption. Such a pair of causal DAG *G* and probability distribution *P* is called causal BN 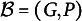 . While a non-causal BN says nothing about the true data-generating mechanism, a causal BN does – first, root nodes (i.e., nodes without direct causes) are generated independently from their marginal distributions, and then recursively, a node is generated from the conditional distribution given its direct causes when all of its direct causes have been generated.

A causal BN entails not only the observational distribution *P* but also distributions subject to various interventions. Formally, let 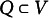 and 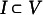 denote the query and intervention sets, and we are interested in calculating the distribution of 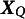 if we set 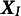 to 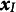 by intervention. Under the Judea Pearl’s *do-calculus* paradigm (Pearl, 1988), that amounts to finding the interventional probability distribution 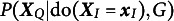 where the do-operator 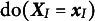 highlights the fact that 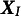 is set to 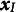 by intervention, not observed to be 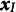 . This interventional probability distribution is generally not equal to the conditional distribution 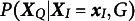 induced from the joint observational distribution 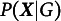 . In fact, 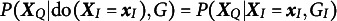 where 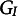 is a “mutilated” version of *G* with all incoming arrows to *I* removed. Intuitively, when there is no intervention, the value of 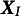 is influenced by its direct causes whereas when 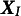 is set to a certain value by intervention, such a value only depends on the intervention^1^. Therefore, in the presence of intervention, the incoming arrows to 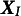 should be removed to reflect the fact that 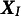 is no longer influenced by its natural causes. Take DAG (t) in Figure 1 as an example. From basic probability theory, the conditional distribution of 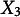 given that 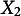 is observed to be 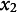 is 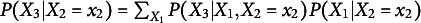 . However, if 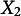 is not naturally observed to be 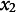 but instead we set its value to 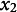 by intervention, the mutilated version of DAG (t) is given by DAG (o) where the arrow from node 1 to node 2 is removed, and the interventional distribution is (2)

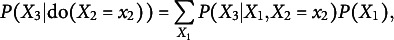

because 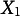 and 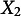 are marginally independent in DAG (o). Being able to derive various interventional distributions using causal BNs is crucial to social and behavior sciences as it does not require real-world interventions, which may be expensive, unethical, or impossible to carry out. For instance, let 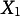 denote age, 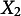 cortical thickness, and 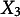 intelligence in the study of the relationship between brain structure and intelligence (Shaw et al., 2006). Suppose the causal relationships of 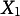 , 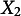 , and 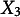 are represented by DAG (t). To identify the average causal effect of cortical thickness on intelligence, i.e., 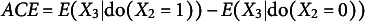 (say, 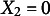 and 1, respectively, represent thin and thick cortex), the gold standard would be to intervene on cortical thickness, which, however, cannot be done. Causal BNs enable such causal effect estimation without carrying out the actual intervention via (2); notice that the right-hand side of (2) does not have the do-operator and hence can be calculated based on observational probability distribution *P* alone.

### 2.4 Learning of causal DAGs and BNs

The preceding paragraphs concern the problem of representation, i.e., given a causal DAG, how one can represent the (stochastic) data-generating mechanism using a probability model. The remaining question is whether one can learn the unknown structure of DAG *G* given a sample generated from the probability model, 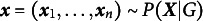 . One intuitive approach is to test for independence. For instance, consider three-node DAGs (there are 25 in total shown in Figure 1), and suppose DAG (h) in Figure 1 is the true data-generating DAG and a large number of observations are available. Assuming causal faithfulness^2^, we sequentially test for independence (i) between 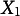 and 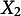 , which comes to be dependent 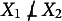 and hence eliminates all DAGs in the blue boxes as they all encode 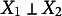 , (ii) between 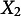 and 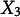 , which comes to be dependent 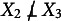 and hence eliminates all DAGs in the green boxes as they all encode 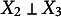 , (iii) between 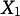 and 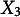 , which eliminates DAG (k) in the yellow box, and (iv) finally between 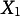 and 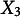 given 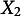 , which comes to be independent 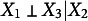 and hence eliminates all DAGs in the purple boxes as they assert dependence 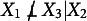 . This example demonstrates that just by applying independence tests on observed data, one can narrow down from 25 possible DAGs to just three DAGs (h-j) that are plausible data-generating mechanisms. This type of approach is called the constraint-based approach. The PC algorithm (Spirtes et al., 2000) is perhaps the most well-known one. However, there are obvious drawbacks of constraint-based approaches: apart from the additional assumption of faithfulness and conditional independence tests generally lacking statistical power, most prominently, they generally can only identify an equivalence class of DAGs, all of which encode exactly the same conditional independence relationships; such DAGs and corresponding BNs are said to be *Markov equivalent* and the equivalence classes are called Markov equivalence classes. In the three-node example, DAGs (h)-(j) have the same set of conditional independencies, i.e., 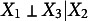 and none other. Therefore, one cannot further narrow it down to the true data-generating DAG (h) even with an infinite sample. This is clearly an unsatisfactory property of constraint-based approaches as DAGs (h)-(j) have very different causal interpretations from each other.

**Figure 2:**
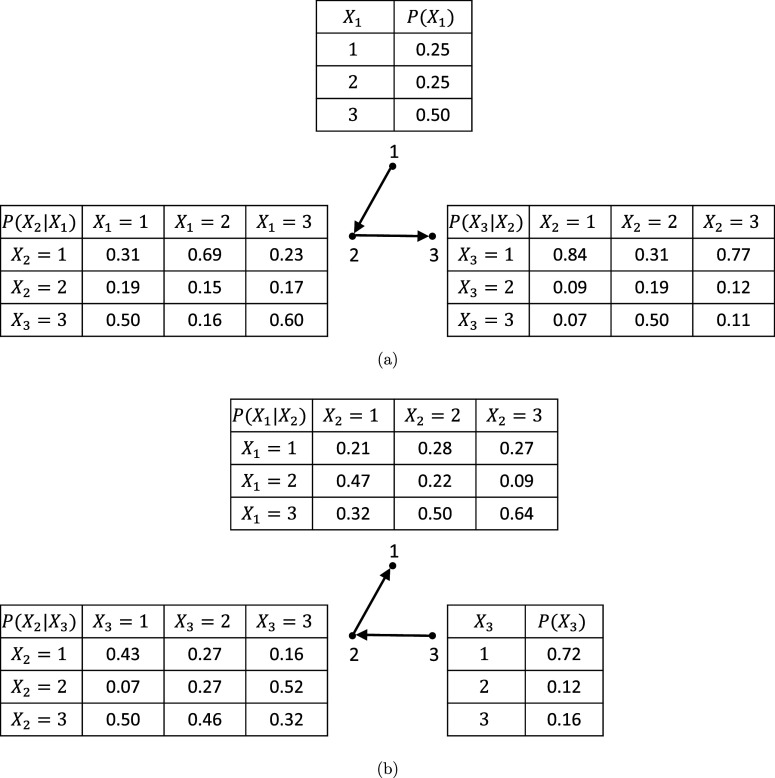
Conditional probability tables from two Markov equivalent BNs.

Another major class of causal DAG learning approaches is score-based where one would assign a score to each DAG and search for highly scored DAGs. Often, the score is based on some probability model and depends on the likelihood. For example, the Bayesian information criterion (BIC) is widely used, (3)

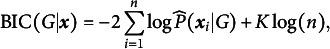

where *K* is the number of model parameters and 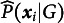 is the joint distribution (1) evaluated at 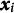 given the maximum likelihood estimate of model parameters. BIC balances between the goodness-of-fit of the causal BN to the observed data and the complexity of the model. For categorical data, 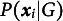 is specified by conditional probability tables as mentioned earlier. Unfortunately, it can be shown that DAGs (h)-(j) are score-equivalent and are, thus, still indistinguishable from each other just like constraint-based methods. We illustrate it with DAGs (h)&(i). Suppose again DAG (h) is the true data-generating DAG and the corresponding conditional probability tables are given in Figure 2(a). These conditional probability tables determine the joint probability distribution of 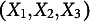 , for example, 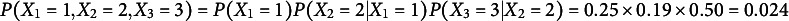 . However, the joint distribution 
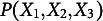
 can be factorized in a few other ways that are also compatible with the conditional independence relationship 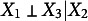 encoded in DAG (h). For example, it can be factorized with respect to DAG (i) in Figure 1 of which the conditional probability tables are shown in Figure 2(b). Consequently, DAGs (h)&(i) have the same BIC score because they have the same maximized likelihood and the same model complexity, and hence cannot be distinguished from each other. This also applies to DAG (j) in Figure 1. More generally, Markov equivalent categorical BNs (cBNs) are (BIC) score-equivalent. Therefore, score-based cBNs cannot differentiate DAGs that are indistinguishable by the constraint-based methods. More discussion of categorical causal BNs is provided in Section 0.6.

We remark that many existing DAG learning algorithms would return a single DAG even though the underlying causal model is not fully identifiable (i.e., there exist equivalent DAGs). Practitioners should be aware that the returned DAG is generally an arbitrary choice from its equivalence class and there may, in fact most likely, exist many other DAGs that fit the data equally well and have very different causal implications. Therefore, we recommend the use of identifiable causal models (e.g., the ordinal BN in the next section) whenever possible.

## 3 Ordinal Bayesian networks

### 3.1 Probability model

Since a lot of questionnaire data in social and behavior sciences are ordinal, we propose the use of ordinal BNs (Ni & Mallick, 2022, oBN) to resolve the indeterminacy of Markov/score-equivalent BNs. Ni and Mallick (2022) theoretically studied the causal identifiability of oBN and showcased its strength in constructing biological networks from observational, discretized protein expression data. oBN can potentially have great utility for discovering causality in questionnaire data. Let 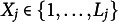 have 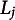 categories for 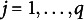 . Each conditional distribution 
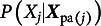
 of (1) takes the form of an ordinal regression model of which the cumulative distribution is given by, for 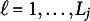 , 

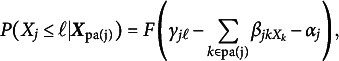

where *F* is a link function such as probit and logistic, 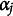 is an intercept, 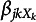 is a generic notation of 
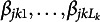
 for 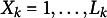 , and 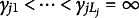 are a set of thresholds. We set 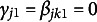 for ordinal regression parameter identifiability (Agresti, 2003). The implied conditional probability distribution is given by, 

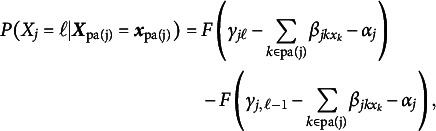

for 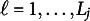 and 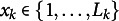 for 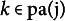 .

To illustrate the identifiability of oBN, consider again the example in Figure 2. Let 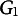 and 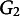 be the DAGs in 2(a) and 2(b), respectively. While a cBN can be factorized in either direction, by exploiting the ordinal nature of categorical data, an oBN does not admit such equivalent factorization. In fact, the conditional probability tables in Figure 2(a) are those under the oBN with DAG 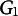 and the following parameter values,


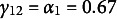
 ,


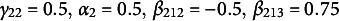
 ,


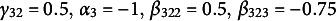
 ,

whereas there are no parameter values of the oBN with DAG 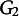 such that the implied observational distribution 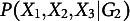 is compatible with the conditional probability tables in Figure 2(b). In other words, 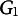 and 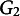 are not score-equivalent as they have different likelihood functions.

### 3.2 Statistical inference

We now develop a score-based DAG learning algorithm, which aims to identify the best-scored DAG with uncertainty quantification. We score each DAG *G* with the BIC (3) where the maximum likelihood estimate is obtained by gradient ascent, and the number of model parameters is 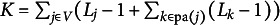 . To search for the best-scored DAG, we use an iterative hill-climbing algorithm. We start from some initial DAG. At each iteration, we score all the DAGs that are reachable from the current graph by a single edge addition, removal, or reversal. We replace the current DAG by the DAG with the largest improvement (i.e., the largest decrease in BIC). We claim the convergence of the algorithm when the BIC can no longer be improved. The hill-climbing algorithm is summarized in Algorithm 1. Since BIC always improves at each iteration by design, the algorithm is guaranteed to find a local optimum.

However, there are two drawbacks of Algorithm 1. First, the local optimum may not be the global optimum due to the greedy nature of the hill-climbing algorithm. Therefore, we suggest repeat the hill-climbing algorithm several times with random initial DAGs and pick the DAG with the smallest BIC as shown in Algorithm 2.

Second, Algorithm 1 or 2 only provides a point estimate of DAG *G* without uncertainty quantification. To assess the uncertainty, we propose to use the bootstrapping technique (Efron, 1992; Friedman et al., 1999). Specifically, we first create a number *B* of bootstrap samples by sampling without replacement from the original data 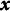 . Then, we apply Algorithm 2 to each bootstrap sample. Finally, we compute the average adjacency matrix of the estimated DAG from each bootstrap sample. An adjacency matrix 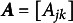 of DAG *G* is a binary matrix such that 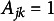 if 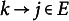 and 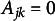 otherwise. Therefore, the average adjacency matrix, denoted by 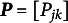 , can be interpreted as an approximate edge inclusion probability of 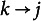 . A value of 
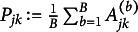
 close to 0 or 1 indicates greater confidence of the absence or presence of 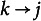 than a value close to 0.5 where the superscript 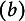 indexes the bootstrap samples. The hill-climbing with multiple initial DAGs and bootstrapping is described in Algorithm 3 and implemented in the R package [name hidden] freely available on CRAN.

### 3.3 Large sample property

Now, we ask if we can correctly identify the data-generating DAG when the sample size is large enough. Let 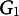 denote the true data-generating DAG with model parameters 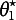 (i.e., 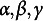 ’s in oBN). Let 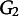 denote any other DAG in the same Markov equivalence class with 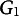 . Let 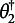 denote its pseudo-true parameter, i.e.,

**Figure:**
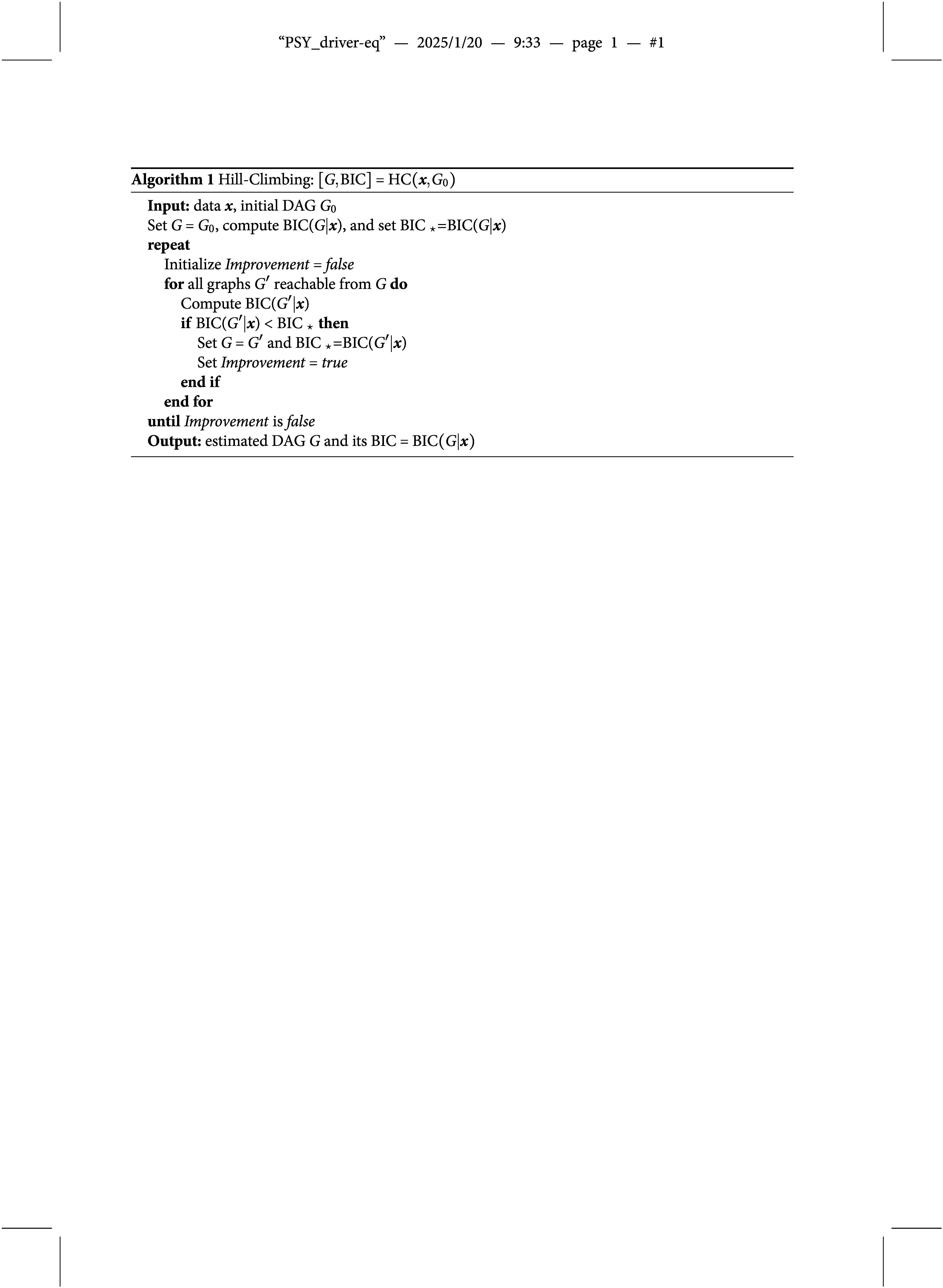

**Figure:**
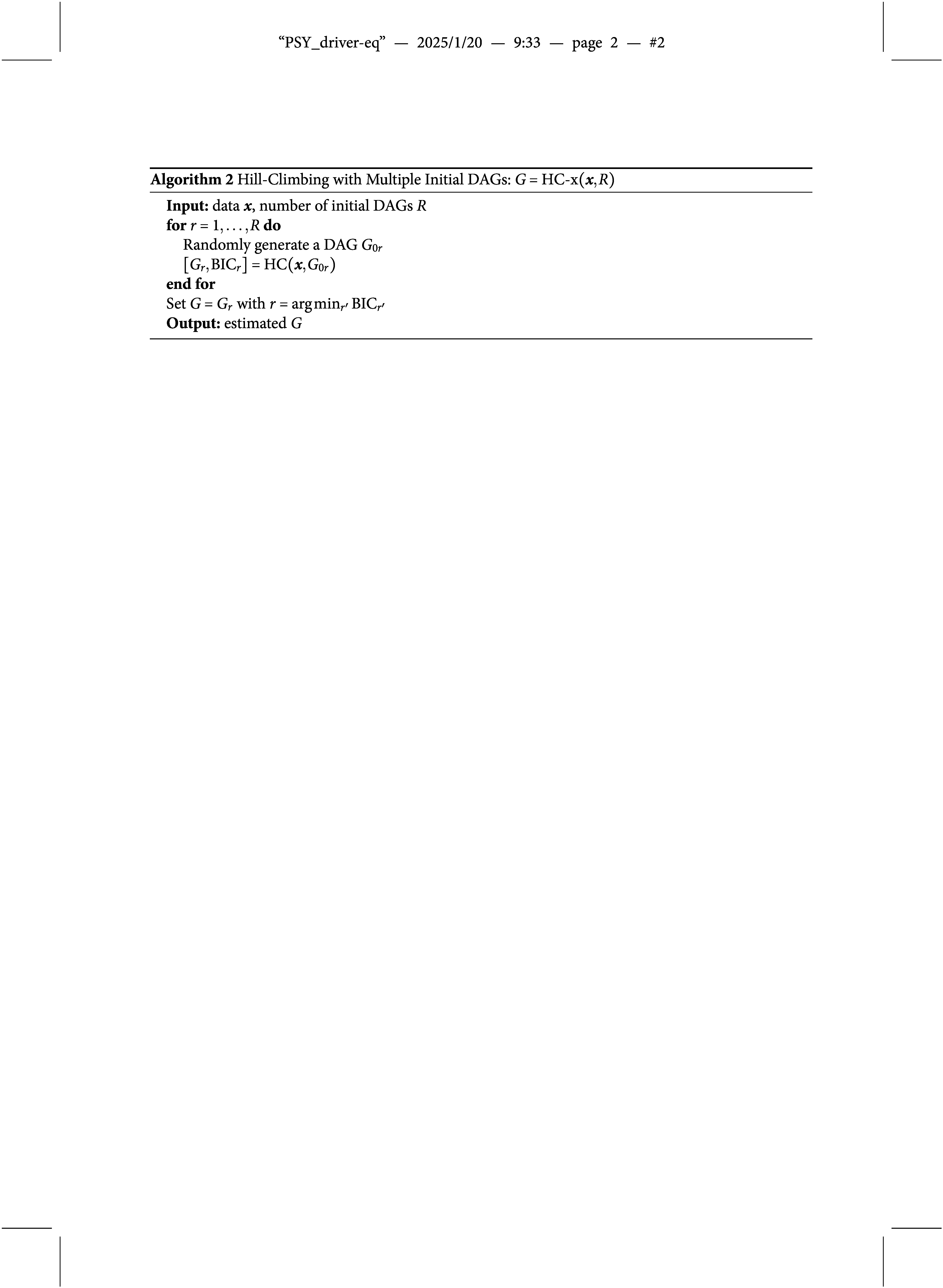

**Figure:**
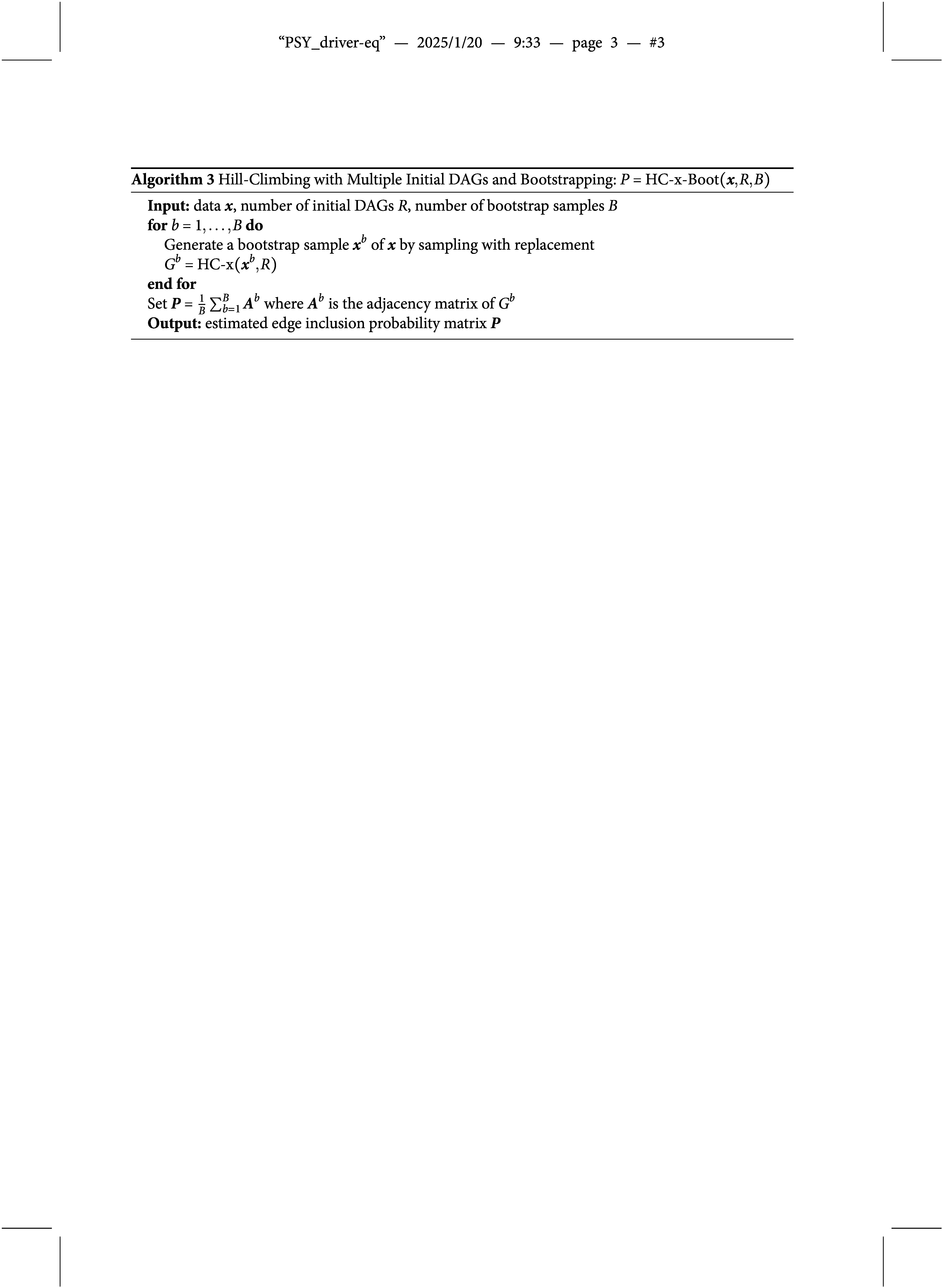




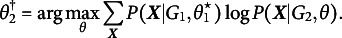



We show that the BIC of 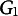 is asymptotically lower than that of 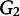 . Let 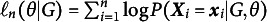 denote the log-likelihood under DAG *G* and let 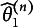 and 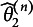 denote the maximum likelihood estimators, which are consistent under some mild regularity conditions (Fahrmeir & Kaufmann, 1985), i.e., 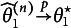 and 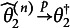 as 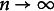 .

Take the Taylor expansion of 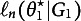 at 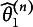 , 

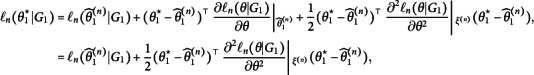

where 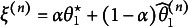 with 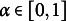 . Since 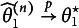 , we have 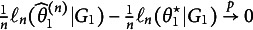 . By the law of large numbers, 

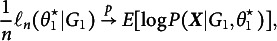

where the expectation is taken over 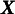 with respect to its true data-generating distribution 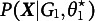 . Therefore, 

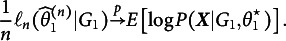

By a similar argument, we have 

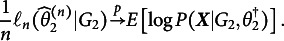

Hence, 

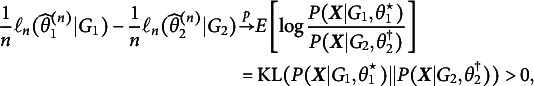

where 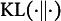 is the Kullback–Leibler divergence, which is nonnegative and is zero only when 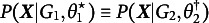 , which is impossible due to the causal identifiability result (Ni & Mallick, 2022). Consequently, 

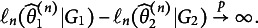

Because two Markov equivalent DAGs must have the same skeleton (Verma & Pearl, 2022) and hence the same model complexity, we have 

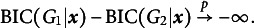



## 4 Simulation studies

**Figure 3:**
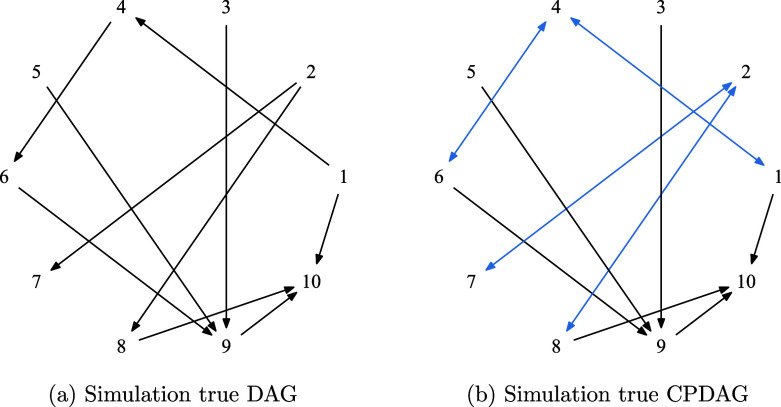
Simulation true (a) DAG and (b) CPDAG. The (blue) bidirected edges in (b) are edges that can be oriented in either direction in the Markov equivalence class represented by the CPDAG.

We assessed the empirical performance of oBN in recovering unknown DAG structure using simulations where the ground truth is known. We simulated data with 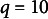 categorical variables each with 5 ordinal categories resembling the 5-point Likert scale questions. The true DAG was generated randomly using the function “randomDAG” in R package pcalg with connecting probability 0.2 (Figure 3a). Its Markov equivalence class, represented by the completed partially directed acyclic graph (CPDAG), is shown in Figure 3b where the bidirected edges are edges that can be oriented in either direction without changing its conditional independence relationships. Given the true DAG, the model parameters 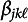 ’s and 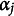 ’s were independently generated from a centered normal distribution with variance 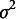 . We considered 14 scenarios. The first 7 scenarios fixed sample size at 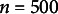 and varied the signal strength 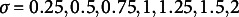 , which covered low to strong levels of signals. The other 7 scenarios fixed the signal strength at 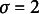 and varied the sample size 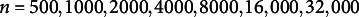 .

**Figure 4:**
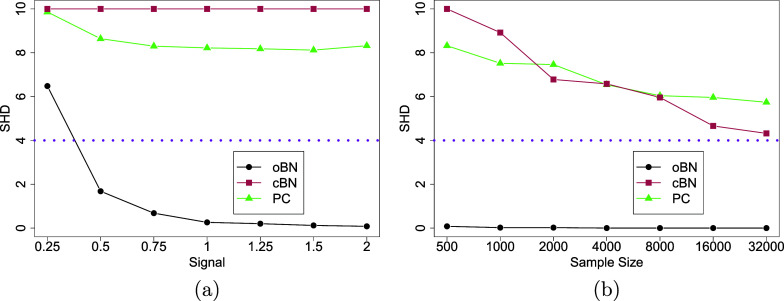
Simulated survey data with ten 5-point Likert scale questions. Panel (a): Sample size 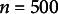 and signal strength varies from 0.25 to 2. Panel (b): Signal strength 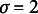 and sample size varies from 500 to 32,000. In both panels, dotted lines indicate the irreducible error (SHD = 4) for an oracle cBN.

**Figure 5:**
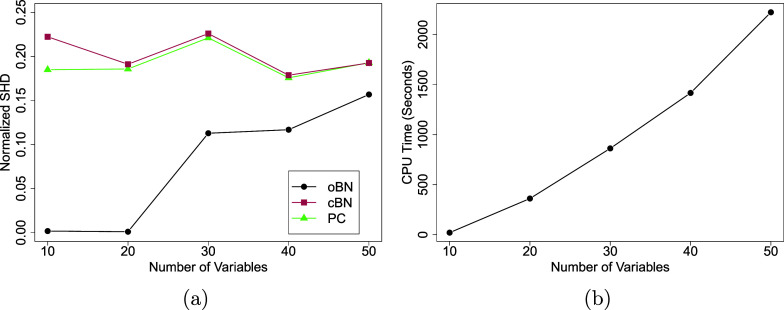
Simulated survey data with varying number of 5-point Likert scale questions 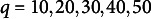 . The sample size is fixed at 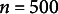 and the signal strength is fixed at 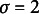 . Left panel: The SHD is normalized by dividing the raw SHD by the total number of edges in a complete DAG (i.e., 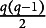 ). Right panel: CPU time of oBN in seconds tested on a 2.9 GHz 6-Core Intel Core i9 CPU.

Algorithm 1, implemented in R package [name hidden], was applied to each simulated dataset. For comparison, we also ran the PC algorithm and the (nominal) cBN. For the PC algorithm, we used a more recent version (Colombo et al., 2014) implemented as “pc.stable()” in the R package bnlearn with the Jonckheere-Terpstra test designed for ordinal data and the type I error controlled at 1%. For the cBN, we used the BIC scoring criterion and the hill-climbing search algorithm with 10 random starts. cBN is also available in the package bnlearn implemented as “hc()”.

As an error measure, we computed the structural hamming distance (SHD) between the estimated graph and the simulation true DAG, which is the number of edge additions, deletions, or reversals required to transform one graph to the other. Note that since cBN and PC can only identify CPDAG (i.e., equivalence classes), the smallest SHD that they can achieve is 4 (the number of bidirected edges in Figure 3b). This error cannot be further reduced for cBN and PC even with an infinite amount of data.

**Table 1:** Sensitivity to the choice of link functions. The average (standard error) SHD is reported

Probit	Logistic	Negative log-log	Complementary log-log
0.26 (0.06)	0.30 (0.07)	0.32 (0.07)	0.28 (0.06)

The SHD averaged over 50 repeat simulations are reported as functions of signal strength 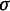 (Figure 4a) and sample size *n* (Figure 4b). Several conclusions can be made. First, because oBN is a fully identifiable model, its SHD quickly approached 0 as signal became stronger; such trend was not observed for cBN and PC. Second, oBN consistently outperformed cBN and PC across all signal levels and sample sizes, which stresses the importance of accounting for the ordinal nature of questionnaire data for causal discovery, which had been overlooked in the literature. Third, when the sample size was moderate 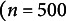 ), the performance of cBN and PC did not improve as the signal strength increased whereas when the signal was strong 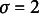 , their performance improved as sample size grew. Eventually, they might reach the irreducible error (SHD = 4 in this example) but that would require a huge amount of data and they cannot do better even with an infinite amount of data. The size of the irreducible error depends on the true data-generating DAG, which could be as large as the total number of edges, which is super-exponential in the number *q* of variables. Fourth, for a large enough sample, oBN perfectly recovered the true DAG, empirically verifying our asymptotic theory.

In summary, our simulation studies suggest that it is advantageous to exploit the ordinal nature of survey questionnaire data for causal discovery. Considering oBN is conceptually similar to cBN but with better theoretical and empirical properties for causal discovery, we hope to see a wider adoption of oBNs in social and behavior sciences. In the following, we conducted additional simulations to test the scalability and sensitivity of our method.

Scalability. We varied the number of variables 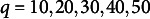 while keeping the sample size at 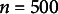 and the signal strength at 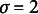 . The data generation process was the same as before. The (normalized) SHD and the CPU time on a 2.9 GHz 6-Core Intel Core i9 CPU averaged over 50 repeat simulations are reported as functions of *q* (Figure 5). As expected, the performance for all methods deteriorated as *q* increased but the proposed oBN still outperformed both cBN and PC, and the computation of oBN scaled reasonably well with *q*.

Sensitivity to link functions. We fitted the proposed model to the data that we simulated earlier (with sample size 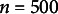 , number of variables 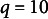 , signal strength 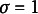 , and the probit link function) using probit, logistic, negative log-log, and complementary log-log link functions. The average SHD between the estimated and true graphs based on 50 repeat simulations is reported in Table 1, which shows our model is relatively robust – the SHDs are well within two standard errors from each other.

## 5 Demonstration: OCD-depression data analyses

To further demonstrate oBN, we analyzed the dataset from a psychological study of the functional relationships between the symptoms of obsessive-compulsive disorder (OCD) and depression (McNally et al., 2017). The dataset consists of 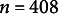 participants’ responses to 10 five-point questions from the Yale-Brown Obsessive-Compulsive Scale via Self-Report (Steketee et al., 1996) measuring the OCD symptoms and 16 four-point questions from the Quick Inventory of Depressive Symptomatology via Self-Report (Rush et al., 2003, QIDS-SR) measuring the depression symptoms. Following Luo et al. (2021), we merged the questions about “decreased appetite” and “increased appetite”, and the questions about “weight loss” and “weight gain” in QIDS-SR since they measure the same depression symptoms. The resulting number of ordinal variables is 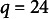 .

**Figure 6:**
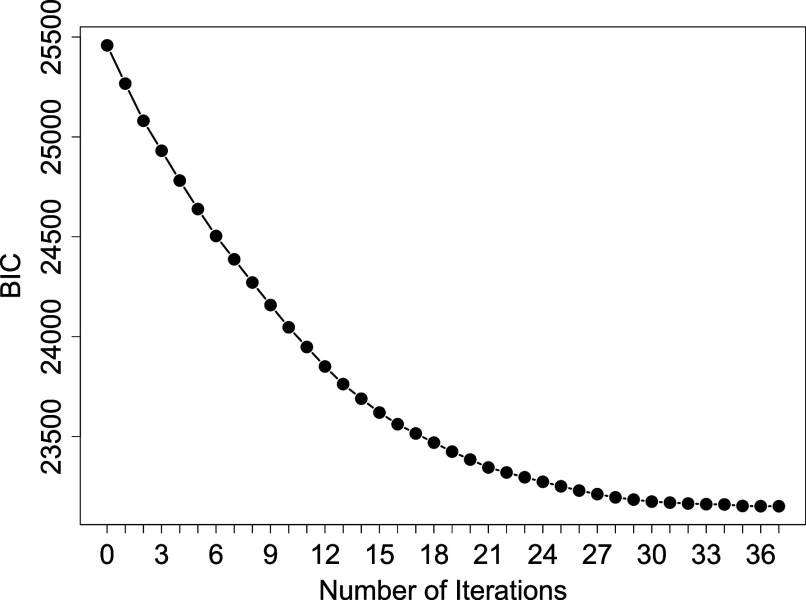
BIC as a function of iteration on the OCD-Depression data.

Algorithm 3 was applied to the dataset with 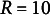 random initial DAGs and 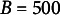 bootstrap samples. For an illustration of the guaranteed convergence, we plot the BIC as a function of iteration in one run of our algorithm in Figure 6, which converged at the 37th iteration.

We also explored a more scalable version of oBN, a two-step hybrid algorithm. Particularly, we first ran the PC algorithm to obtain a CPDAG, and then, for any pair of nodes with an undetermined edge (i.e., the edge can be oriented in either direction), we ran the bivariate version of oBN to determine its direction; the pseudocode is presented in Algorithm 4.

**Figure:**
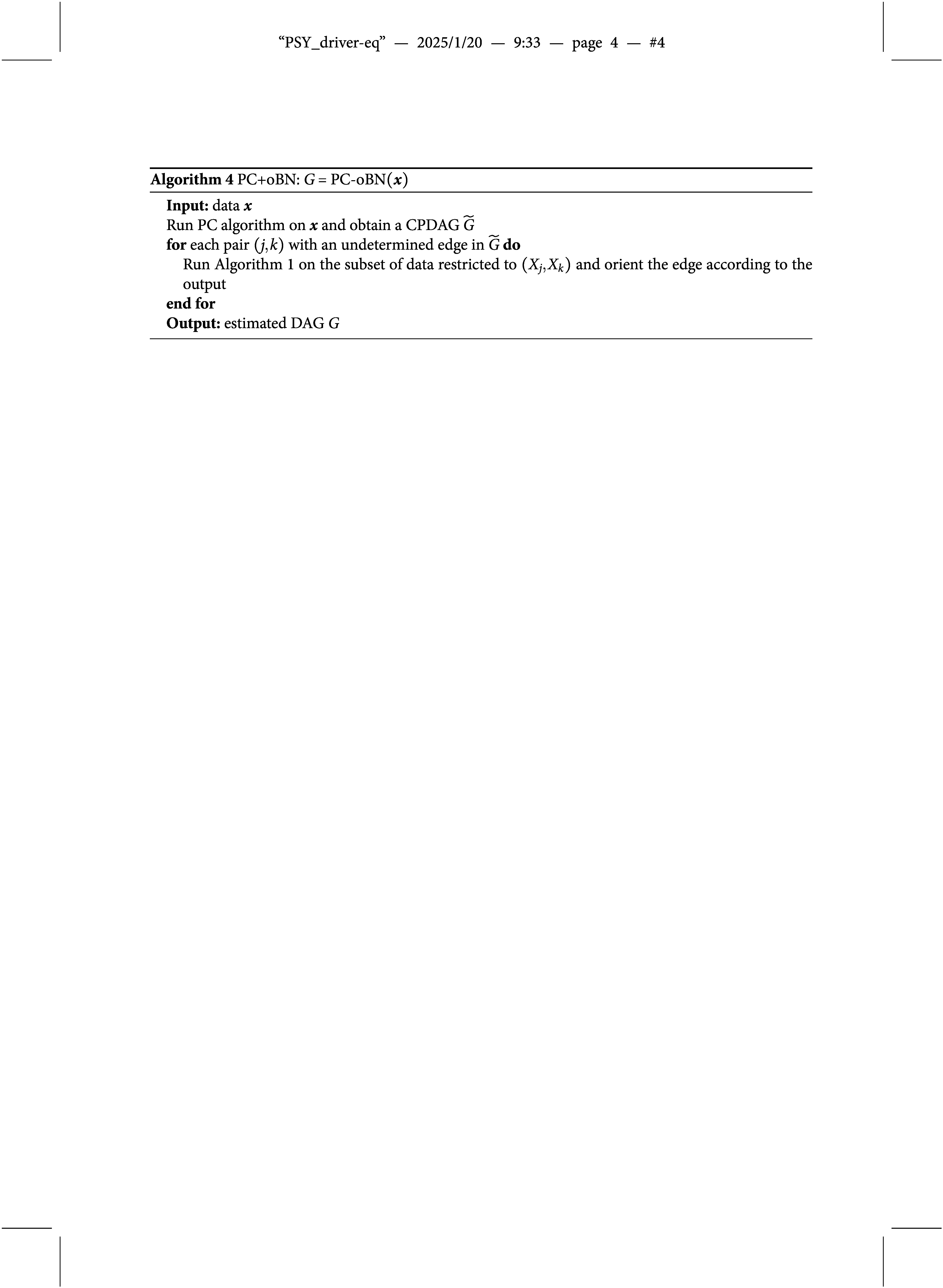

**Figure 7:**
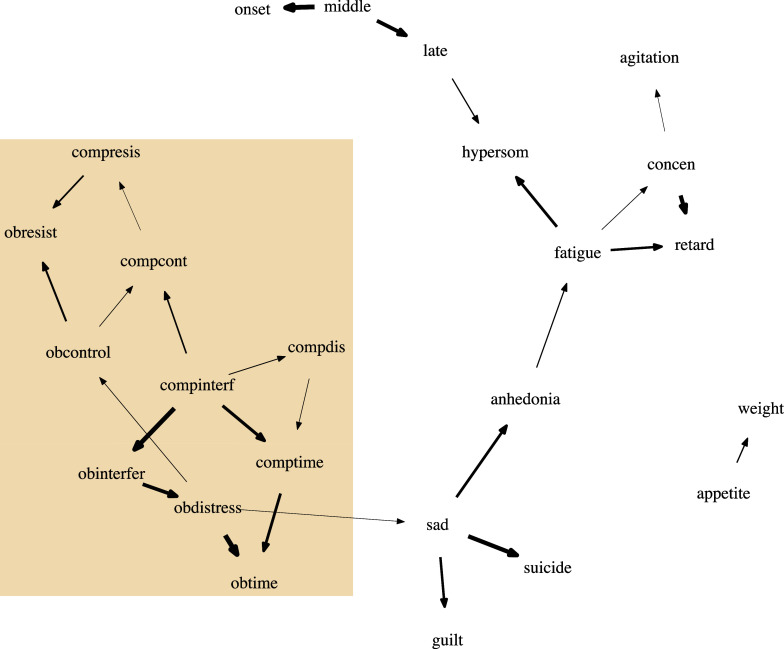
Estimated OCD-Depression networks using oBN with 500 bootstrap samples. The edge width is proportional to its probability. Nodes within the box are the ten OCD-related variables.

**Figure 8:**
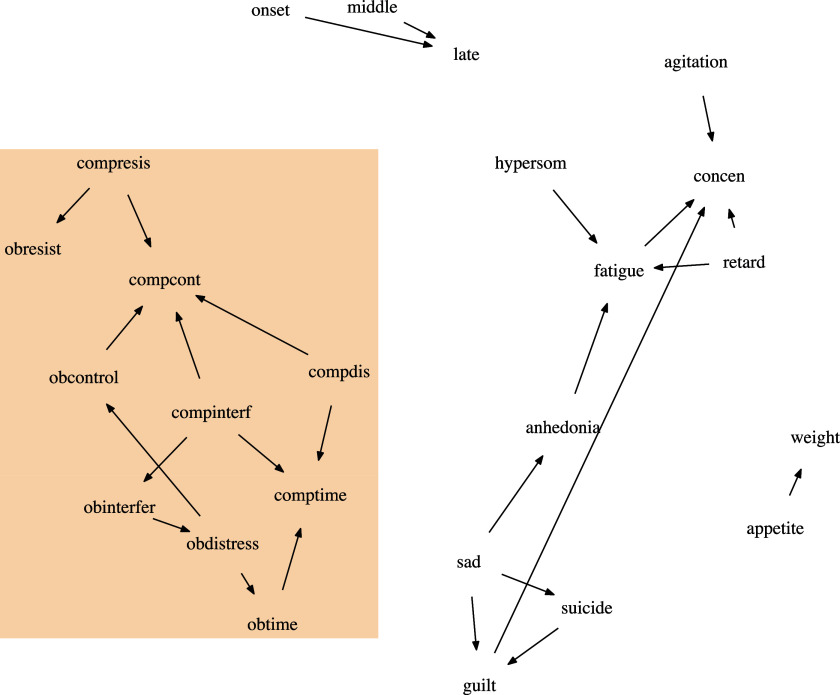
Estimated OCD-Depression networks using PC+oBN. Nodes within the box are the ten OCD-related variables.

**Figure 9:**
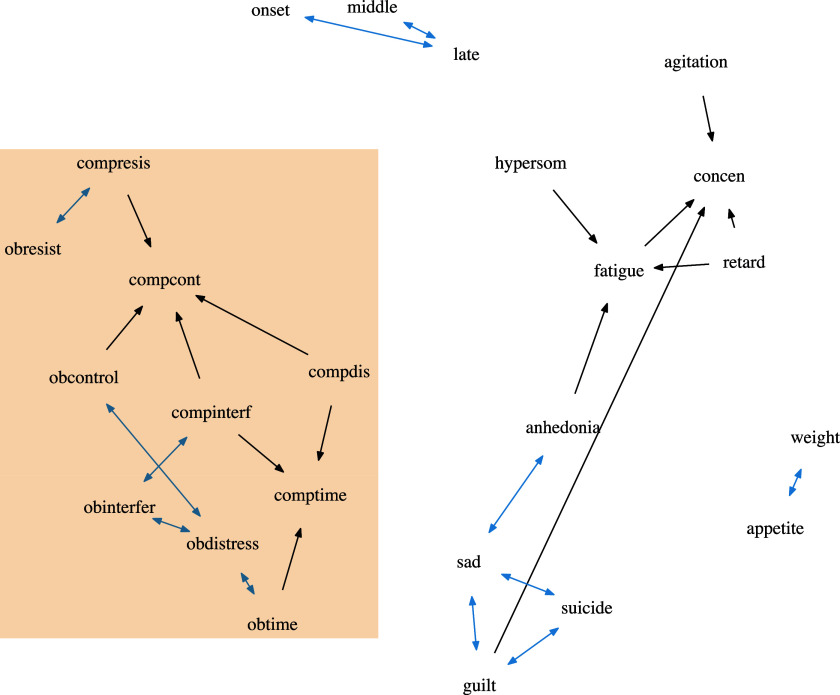
Estimated OCD-Depression networks using PC. The (blue) bidirected edges are edges of which the directionality is undetermined. Nodes within the box are the ten OCD-related variables.

**Figure 10:**
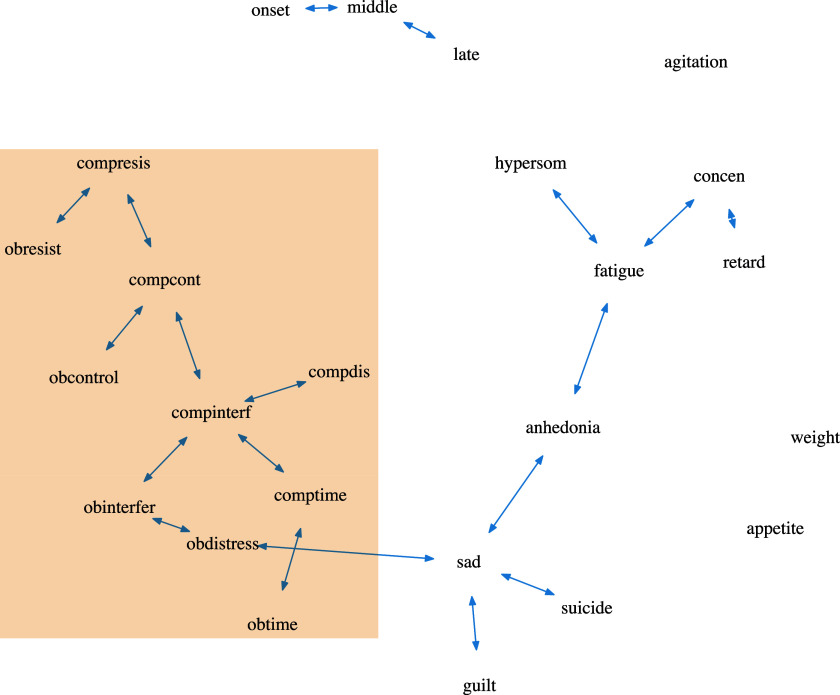
Estimated OCD-Depression networks using cBN with BIC and hill-climbing. The (blue) bidirected edges are edges of which the directionality is undetermined. Nodes within the box are the ten OCD-related variables.

**Figure 11:**
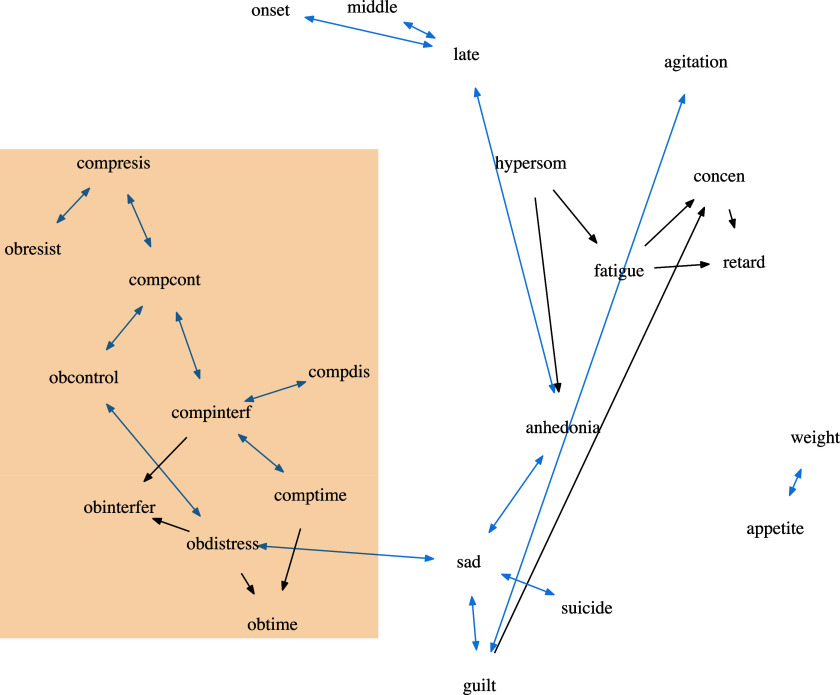
Estimated OCD-Depression networks using OSEM. The (blue) bidirected edges are edges of which the directionality is undetermined. Nodes within the box are the ten OCD-related variables.

In Figure 7, we plot the estimated DAG from oBN with edge width proportional to the inclusion probability; also see the list of all the significant edges (i.e., 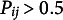 ) ranked by their inclusion probabilities in Table 2. For brevity, we have adopted the same abbreviation of the symptoms as in McNally et al. (2017). Comparing to the estimated network by PC+oBN (Figure 8), all the undetermined edges from the PC algorithm (Figure 9) were oriented consistently between oBN and PC+oBN. For comparison, we also applied the PC algorithm with the Jonckheere-Terpstra test, the cBN with BIC and hill-climbing, and the ordinal structural equation model (Luo et al., 2021, OSEM) with the caveat that we do not know the underlying true causal relationships. Their results are reported in Figures 9-11. Some interesting observations can be made.

Common to all the methods, the symptoms of OCD and the symptoms of depression were found to be largely separated meaning that most of the symptoms of OCD do not directly cause most of the symptoms of depression, and vice versa. This is perhaps not surprising given that OCD and depression are different psychological disorders. oBN, cBN, and OSEM did simultaneously find one bridge causal link between OCD (*obdistress*) and depression (*sad*). The existence of a bridge causal link is plausible because many studies have suggested that more than one third of OCD patients have concurrent depression (Abramowitz, 2004; Hong et al., 2004; Nestadt et al., 2001). cBN and OSEM, due to their non-identifiability, could not determine the direction of that causal link whereas oBN identified it to be *obdistress* 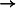 *sad*. This again seems to agree with existing studies that OCD symptoms often precede depression in individuals who suffer from both disorders (Anholt et al., 2011; Meyer et al., 2014; Zandberg et al., 2015). Our finding suggests that controlling distress caused by obsession may help alleviate or prevent depression symptoms.

**Table 2:** OCD-Depression data. A list of significant edges identified by oBN ranked by inclusion probabilities

Significant edge			Probability
Obdistress	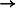	Obtime	0.98
Middle	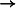	Onset	0.972
Compinterf	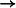	Obinterfer	0.946
Sad	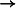	Suicide	0.914
Concen	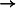	Retard	0.898
Middle	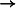	Late	0.862
Obinterfer	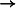	Obdistress	0.846
Compinterf	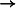	Comptime	0.79
Fatigue	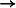	Hypersom	0.758
Comptime	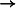	Obtime	0.756
Sad	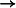	Anhedonia	0.748
Sad	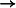	Guilt	0.722
Fatigue	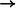	Retard	0.716
Obcontrol	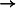	Obresist	0.67
Compresis	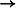	Obresist	0.636
Compinterf	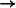	Compcont	0.624
Appetite	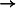	Weight	0.596
Late	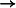	Hypersom	0.588
Anhedonia	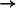	Fatigue	0.58
Compinterf	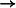	Compdis	0.554
Obdistress	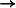	Obcontrol	0.53
Obcontrol	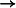	Compcont	0.53
Fatigue	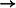	Concen	0.528
Compdis	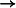	Comptime	0.524
Obdistress	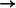	Sad	0.514
Concen	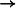	Agitation	0.508
Compcont	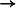	Compresis	0.504

Within the symptoms of OCD, on the one hand, the links among obsessive symptoms and the links among compulsive symptoms are the links that tend to have the highest probabilities, e.g., *obinterfer* 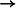 *obdistress* (0.846), *obdistress* 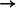 *obtime* (0.98), and *compinterf* 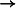 *comptime* (0.79). This matches the hypothesized two dimensions of obsession and compulsion in theoretical models (de Wildt et al., 2005). On the other hand, there also exist significant links from compulsion symptoms to their obsession counterparts, including *compinterf* 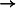 *obinterfer*, *comptime* 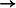 *obtime*, and *compresis* 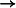 *obresist*, which are qualitatively consistent with previous network analyses (Carbonella, 2018; Cervin et al., 2020), although their network models are undirected/non-causal. According to our results, one can potentially suppress obsession symptoms by suppressing the corresponding compulsion symptoms but not vice versa.

Within the symptoms of depression, the link between *sad* and *suicide* was found by all the methods but only oBN and PC+oBN were able to determine its direction *sad* 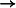 *suicide* (inclusion probability = 0.914 for oBN). It is well-known that persistent feeling of sadness is a major risk factor for suicide (Angst et al., 1999; Brådvik, 2018). Another expected link was *appetite* 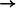 *weight*, of which the direction was again only identified by oBN and PC+oBN.

In summary, although the ground truth is not available, our data analyses, in our opinion, support the usefulness of oBN for generating plausible psychological hypotheses in practice.

## 6 Discussion

We have demonstrated the functionality of oBNs as a useful alternative to the potential outcome framework and SEMs for analyzing survey questionnaire data. oBNs can provide an unbiased causal view of a complex system without any prior structural knowledge. Moreover, unlike other existing BNs for categorical data, oBNs are fully identifiable with observational data alone.

Note that both cBN and oBN utilize the BIC score. Therefore, the difference between cBN and oBN lies in the likelihood function and the model complexity. For oBN, the likelihood function is specified by ordinal regression whereas for the cBN, it is specified by multinomial distribution. The model complexity is different between the two methods, 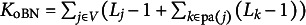 for oBN and 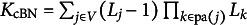 for cBN.

There also exist copula-based methods (Castelletti, 2024; Cui et al., 2016), which are quite flexible in incorporating different types of data including ordinal data. While the estimation procedures are all very different from each other, the main theoretical difference between the proposed oBN model and the copula-based methods is three-fold:

1. The learned causality or conditional independence is on the observed variable level for the proposed oBN model and is on the latent variable level for the copula-based methods. For discrete variables, the latter is not equivalent to the former.
2. For mixed data, the proposed oBN model needs to discretize the count/continuous data whereas copula-based methods do not need to because of the latent continuous variable representation.
3. The proposed oBN is uniquely identifiable whereas the copula-based methods are only identifiable up to the Markov equivalence class.

The proposed causal structure learning algorithms based on hill-climbing and bootstrapping worked quite well in simulation studies and also generated some plausible causal hypotheses in the real data. Although this paper focuses on causal structure learning, the discovered DAG structure can be used to determine the causal effects. Following Castelletti et al. (2023), we define the causal effect of 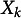 on 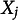 at level 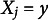 and 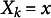 using 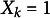 as the reference level for 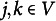 , 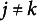 , 
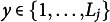
 , and 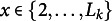 as,

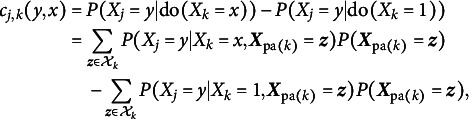

where 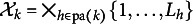 . Given the estimated DAG and model parameters, the conditional/marginal probabilities needed to compute the causal effect can be calculated using the sum-product message passing algorithm (Koller & Friedman, 2009, Chapter 10).

We hope we have convinced researchers to start using oBNs instead of cBNs or PC for ordinal questionnaire data. There are, of course, limitations of oBNs. First, feedbacks are not allowed in BNs. This may partially explain the inconsistency of some of the causal directions (e.g., the link between *fatigue* and *hypersom*) across the methods in the OCD-Depression data analyses. To infer feedbacks, directed cyclic graphical models may be used. However, we are not aware of the existence of such model for categorical data. Second, the learning algorithm has no guarantee for global convergence. Although Algorithm 2 with multiple random initializations can help mitigate this issue, a more principled solution would be via Bayesian causal structure learning algorithms (Choi et al., 2020), which have theoretical convergence guarantees. We leave it as future work since the proposed algorithms in this paper already showed empirically favorable performance compared to alternative methods. Third, the identifiability theory of Ni and Mallick (2022) requires the causal sufficiency assumption, i.e., there is no unmeasured confounder, of which the validity is difficult to check in practice. Fortunately, their sensitivity analyses provide some assurance that oBN is reasonably robust with respect to the presence of unmeasured confounders. Fourth, oBNs are exploratory, not confirmatory. To confirm the causal hypotheses generated from oBNs, one has to, ultimately, resort to clinical interventions, which is beyond the scope of this paper.

## Footnotes

1 Such intervention is also known as the hard intervention in causal discovery literature.

2 A distribution *P* is said to be faithful to a causal DAG *G* if all conditional independence relationships of *P* are encoded in *G* through its Markov property.
